# Infective Endocarditis Hospitalizations and Outcomes in Patients With End‐Stage Kidney Disease: A Nationwide Data‐Linkage Study

**DOI:** 10.1161/JAHA.121.022002

**Published:** 2021-09-28

**Authors:** Peter J. Gallacher, David A. McAllister, Nicholas L. Mills, Nicholas L. Cruden, Anoop S. V. Shah, Neeraj Dhaun

**Affiliations:** ^1^ BHF/University Centre for Cardiovascular Science University of Edinburgh United Kingdom; ^2^ Usher Institute University of Edinburgh United Kingdom; ^3^ Institute of Health and Wellbeing University of Glasgow United Kingdom; ^4^ Edinburgh Heart Centre Royal Infirmary of Edinburgh United Kingdom; ^5^ London School of Hygiene and Tropical Medicine London United Kingdom; ^6^ Department of Renal Medicine Royal Infirmary of Edinburgh United Kingdom

**Keywords:** end‐stage renal disease, epidemiology, infective endocarditis, Cardiovascular Disease, Epidemiology

## Abstract

**Background:**

We investigated the clinical features, microbiology, and short‐ and long‐term outcomes of incident infective endocarditis (IE) hospitalizations in patients with end‐stage kidney disease (ESKD) requiring dialysis or with a kidney transplant over 25 years in Scotland.

**Methods and Results:**

In this retrospective, population‐based cohort study linking national hospitalization and mortality data, we identified patients with a history of ESKD and hospitalized with IE in Scotland between January 1, 1990 and December 31, 2014. From January 1, 2008, individual IE hospitalizations were additionally linked to national microbiology data. Multivariable logistic regression, adjusting for patient demographics and comorbidities, evaluated the association between ESKD and all‐cause death at 1 and 3 years. Of 7638 incident IE hospitalizations between 1990 and 2014, 2.8% (216/7638) occurred in 210 patients with ESKD and 97.2% (7422/7638) occurred in 7303 patients without ESKD. Positive findings from blood cultures were identified in 42% (950/2267) of incident IE hospitalizations from 2008. *Staphylococcus aureus* was isolated in 25.9% (21/81) and 12.8% (280/2186) of patients with and without ESKD, respectively (*P*=0.002). ESKD was associated with an increased odds of death at 1 (44.9% versus 31.4%; adjusted odds ratio [aOR], 2.47, 95% CI, 1.85–3.30;, *P*<0.001) and 3 years (63.9% versus 42.8%; aOR, 3.77; 95% CI, 2.79–5.12; *P*<0.001).

**Conclusions:**

IE is associated with a poor prognosis in patients with ESKD, especially in the longer term. Compared with patients without ESKD, patients with ESKD were twice as likely to die within 1 year, and 3 times as likely to die within 3 years of IE hospitalization.

**Nonstandard Abbreviations and Acronyms**
ESKD: end‐stage kidney disease
IE: infective endocarditis
SIMD: Scottish Index of Multiple Deprivation

Infectious diseases are the second commonest cause of death after cardiovascular disease in patients with end‐stage kidney disease (ESKD) requiring dialysis or with a kidney transplant.^1^, ^2^ The incidence of infective endocarditis (IE) is ≈50‐ to 70‐fold higher in those with ESKD compared with the general population, partly attributable to the use of arterio‐venous grafts and indwelling catheters in patients undergoing dialysis, and long‐term immunosuppression in renal transplant recipients.^2^, ^3^, ^4^ The limited data available—mostly from small or single‐center studies with short follow‐up—suggest that the prognosis of IE in patients with ESKD is poor.^2^, ^3^

With the increasing global burden of ESKD,^5^ contemporary population‐based studies detailing the burden and outcomes of IE in patients with ESKD would be invaluable for planning healthcare provision for this at‐risk group. The aim of this nationwide data linkage study was to investigate the clinical features, microbiology and long‐term outcomes of incident IE hospitalizations in patients with and without ESKD over the past 25 years in Scotland.

## METHODS

### Study Design

We conducted a retrospective, population‐based cohort study linking national hospitalization, microbiology, and mortality data sets in Scotland (Data S1).^4^

### Study Population

Using *International Classification of Diseases* (*ICD*) coding and a 5‐year look‐back period (Tables S1 and S2), incident IE hospitalizations were identified from national inpatient records in those aged ≥20 years admitted to any Scottish hospital between January 1, 1990 and December 31, 2014. To optimize specificity and sensitivity, we included only hospitalizations with a diagnostic code for IE appearing in the first 2 (of 6) positions of the national inpatient record. We extracted demographic data (age, sex, and deprivation status) and selected comorbidities (history of stroke, heart failure, myocardial infarction, cardiac device, and previous cardiac valvular surgery) (Data S1). Patients with ESKD were identified by searching linked inpatient records before incident IE hospitalization for relevant *ICD* codes (Table S1) appearing in any of the 6 available diagnostic positions. Additionally, between January 1, 2008 and December 31, 2014, incident IE hospitalizations were linked to blood culture data obtained from national microbiology records (Data S1).

### Determination of Social Deprivation Status and Comorbidities

Social deprivation status was determined according to the Scottish Index of Multiple Deprivation (SIMD) (Data S1).^6^ SIMD is a geographical‐based measure of deprivation. It is measured in quintiles, where the first and fifth quintiles are the most and least deprived, respectively. Every patient was assigned an SIMD quintile based on their individual SIMD rank at the time of incident IE hospitalization—determined by social factors related to residential address (zip code). Comorbidities (history of myocardial infarction, stroke, heart failure hospitalization, implanted cardiac device, and prior valvular heart surgery) were defined by identifying relevant *ICD* codes attributed to inpatient records of hospitalizations and procedures during the 5 years preceding incident IE hospitalization (Data S1).^4^

### Study Outcomes

Outcomes included stroke, heart failure, and subsequent valvular heart surgery at 1 and 3 years; and all‐cause death at 30 days, 1 year, and 3 years.

### Statistical Analysis

Clinical characteristics and outcomes were summarized according to ESKD status. Groupwise comparisons were performed using Chi‐square tests, as appropriate. Multivariable logistic regression (adjusted for age, sex, social deprivation status; history of stroke, heart failure and myocardial infarction) evaluated the association between ESKD and all‐cause death at 1 and 3 years in all patients hospitalized with IE. In a within‐exposure analysis restricted to those with ESKD, multivariable logistic regression was used to determine factors associated with all‐cause death at 1 and 3 years. Age, sex, and outcome data were complete for all IE hospitalizations. Data on social deprivation status were missing in 0.6% of all IE hospitalizations; these records were excluded from our analysis. Statistical analysis was performed in R, Version 3.5.1 (Vienna, Austria).

### Ethical Considerations and Access to Study Data

The study was approved by the National Health Service Public Benefit and Privacy Panel (reference: 1516‐0116). Patient consent was not sought as the analysis used fully anonymized data. Individual‐level data are available via application to the National Health Service e‐Data Research and Innovation Scotland team, which is part of Public Health Scotland. Access to original study data are restricted to approved members of the research team and will not be made publicly available. However, these individual‐level data are available via application to the National Health Service e‐Data Research and Innovation Scotland team, which is part of Public Health Scotland. Source analysis code will be made available upon request to the corresponding author.

## RESULTS

Of 7638 incident IE hospitalizations between January 1, 1990 and December 31, 2014, 2.8% (216/7638) occurred in 210 patients with ESKD and 97.2% (7422/7638) occurred in 7303 patients without ESKD. Patients with ESKD were younger (58.9±14.3 versus 65.2±17.3 years) and more likely to be men (56.9% versus 48.4%) than patients without ESKD (Table). Between January 1, 2008 and December 31, 2014, there were a total of 950 (41.9%) positive blood cultures associated with 2267 incident IE hospitalizations (Table S3). During this period, the rate of positive blood cultures was higher in patients with ESKD than in patients without ESKD (58% [47/81] versus 41.3% [903/2186]) (*P*<0.004).

**Table 1:** Clinical Characteristics and Outcomes of Patients With and Without ESKD Hospitalized With Incident Infective Endocarditis in Scotland Between 1990 and 2014

	ESKD	No ESKD
No. of hospitalizations, n (%)	216 (2.8)	7422 (97.2)
Mean age, y (SD)	58.9 (14.3)	65.2 (17.3)
Sex, n (%)		
Men	123 (56.9)	3595 (48.4)
Women	93 (43.1)	3827 (51.6)
SIMD quintile, n (%)*		
1 (most deprived)	49 (22.7)	1878 (25.3)
2	56 (25.9)	1687 (22.7)
3	51 (23.6)	1386 (18.7)
4	27 (12.5)	1273 (17.2)
5 (least deprived)	31 (14.4)	1156 (15.6)
Previous medical conditions, n (%)		
Myocardial infarction	14 (6.5)	339 (4.6)
Stroke	16 (7.4)	400 (5.4)
Heart failure	24 (11.1)	1141 (15.4)
Cardiac device	9 (4.2)	199 (2.7)
Prior cardiac valve surgery	18 (8.3)	679 (9.1)
All‐cause death, n (%)		
30 d	40 (18.5)	1041 (14.0)
1 y	97 (44.9)	2329 (31.4)
3 y	138 (63.9)	3179 (42.8)
Other outcomes at 1 y, n (%)		
Stroke	13 (6.0)	310 (4.2)
Heart failure hospitalization	16 (7.4)	914 (12.3)
Subsequent cardiac valve surgery	30 (13.9)	783 (10.5)
Other outcomes at 3 y, n (%)		
Stroke	14 (6.5)	443 (6.0)
Heart failure hospitalization	21 (9.7)	1272 (17.1)
Subsequent cardiac valve surgery	34 (15.7)	942 (12.7)

ESKD indicates end‐stage kidney disease; and SIMD, Scottish Index of Multiple Deprivation.

* SIMD quintile was available for 99.1% (214/216) and 99.4% (7,380/7,422) of patients with and without ESKD, respectively.

*Staphylococcus* spp. was the commonest genus identified in patients with (35.8%, 29/81) and without ESKD (17.1%, 374/2186) (*P*<0.001) (Table S3). *Staphylococcus aureus* was isolated in 25.9% (21/81) of patients with ESKD compared with 12.8% (280/2186) of patients without ESKD (*P*=0.002), whilst coagulase‐negative *Staphylococcus* spp. were identified in 9.9% (8/81) of patients with ESKD versus 4.3% (94/2186) of patients without ESKD (*P*<0.03). In contrast, *Streptococcus* spp. was observed in 9.9% (8/81) of patients with ESKD and 15.1% (329/2186) of patients without (*P*=0.264).

Within 1 year of incident IE hospitalization, 13.9% (30/216) of patients with ESKD and 10.5% (783/7422) of patients without ESKD underwent valvular heart surgery (Table). At 3 years, these figures were 15.7% (34/216) and 12.7% (942/7422), respectively. All‐cause mortality at 30 days was 18.5% (40/216) in patients with ESKD, compared with 14.0% (1041/7422) in patients without ESKD (Table). Within 1 year of incident IE hospitalization, 44.9% (97/216) of patients with ESKD and 31.4% (2329/7422) of patients without ESKD had died (adjusted odds ratio [aOR], 2.47; 95% CI, 1.85–3.30; *P*<0.001) (Figure [A]). At 3 years, these figures were 63.9% (138/216) and 42.8% (3179/7422), respectively (aOR, 3.77; 95% CI, 2.79–5.12; *P*<0.001) (Figure B).

**Figure 1:**
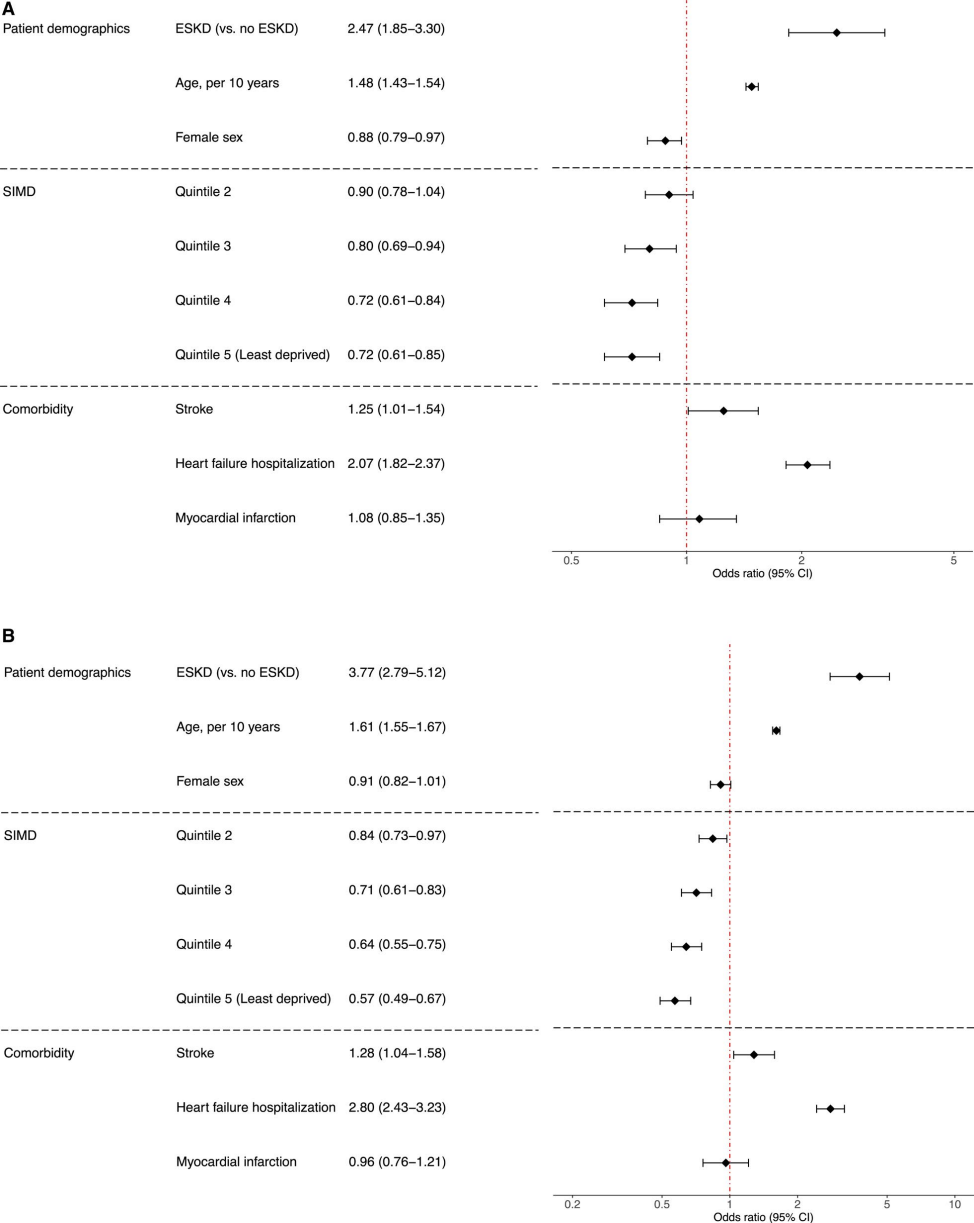
Forest plot showing adjusted odds ratios and 95% CIs from multivariable logistic regression models evaluating the association between end‐stage kidney disease and all‐cause death at 1 (**A**) and 3 years (**B**). Number of observations (each model): 7594. Please note x‐axes for (A and B) have different scales. ESKD indicates end‐stage kidney disease; and SIMD, Scottish Index of Multiple Deprivation.

In patients with ESKD, older age was associated with a significantly increased odds of death at both 1 (aOR, 1.34 per 10‐year increase in age; 95% CI, 1.09–1.67; *P*<0.001) and 3 years (aOR, 1.44 per 10‐year increase in age; 95% CI, 1.16–1.81; *P*=0.001) following incident IE hospitalization. However, lower levels of social deprivation were associated with a reduced odds of death at 3 years only (aOR, 0.34 for least versus most deprived SIMD quintile; 95% CI, 0.12–0.92; *P*=0.037) (Table S4).

## DISCUSSION

To date, this is one of the largest nationwide data linkage studies to compare the clinical characteristics, microbiology, and long‐term outcomes of IE in patients with and without ESKD. One fifth of patients with ESKD died within 30 days of their incident IE hospitalization, half died within 1 year, and two thirds within 3 years. Compared with those without ESKD, patients with ESKD were twice as likely to die within 1 year of IE hospitalization and >3 times as likely to die within 3 years. In patients with ESKD, older age was independently associated with a poorer prognosis at both 1 and 3 years, whilst the lowest level of deprivation was associated with better outcomes at 3 years only.

The few studies that have defined the microbiology of IE in patients with ESKD report *Staphylococcus aureus* infection rates of ≈50% to 80%^7^, ^8^—substantially higher than in the current study. However, these types of cohort study often rely on cases of IE being identified by clinicians, increasing the risk of selection bias and thus, the proportion of patients with positive microbiology. In contrast, our study—which used individual patient‐level blood culture data—was free from selection bias as all IE hospitalizations were identified from routine diagnostic coding, the accuracy of which was recently reported as ≈94% for cardiovascular diagnoses.^9^

Whilst short‐term outcomes of IE are similar between those with and without ESKD, patients with ESKD do worse in the longer term. Our data are consistent with a contemporary Danish study^3^ and compare favorably with an American study in patients with ESKD performed ≈20 years ago,^10^ which described 1‐ and 3‐year mortality rates of 61.6% and 81.7%, respectively. In addition, the in‐hospital mortality described in a recently published single‐center study from Taiwan was approximately double the figure we report at 30 days, despite similar *Staphylococcus aureus* infection rates.^11^ Bhatia and colleagues^12^ previously demonstrated a comparable increased risk of death in patients with and without ESKD. Although well‐powered, their analysis was restricted to in‐hospital death only. In this regard, the long‐term outcomes we report are more relevant in the contemporary era, given ≈80% of patients with ESKD survive the initial IE hospitalization.

There are some limitations to consider. Our analysis was under‐powered to stratify outcomes by renal replacement therapy (hemodialysis, peritoneal dialysis or renal transplant) or microbiological etiology, or to include these variables in the multivariable regression analyses. As we used routine administrative *ICD* codes to identify IE hospitalizations, our study was free from selection bias but subject to case ascertainment bias. To limit the impact of this, the study cohort was restricted to hospitalizations with a diagnostic code for IE in the first 2 (of 6) positions.

Overall, this comprehensive nationwide analysis over a 25‐year period highlights the poor prognosis associated with IE in patients with ESKD compared with patients without ESKD, especially in the longer term. Our results underscore the importance of a multidisciplinary approach to the clinical management of this complex and vulnerable patient group.

## Sources of Funding

Gallacher is supported by a Medical Research Grant from the Mason Medical Research Foundation and a Clinical Research Training Fellowship (FS/CRTF/20/24079) from the British Heart Foundation. Shah is supported by an Intermediate Clinical Research Fellowship (FS/19/17/34172) from the British Heart Foundation. Mills is supported by a Senior Clinical Research Fellowship (FS/16/14/32023) and a Research Excellence Award (RE/18/5/34216) from the British Heart Foundation. Dhaun is supported by a Senior Clinical Research Fellowship from the Chief Scientist Office (SCAF/19/02).

## Disclosures

Shah has received honoraria from Abbott Diagnostics. Mills has received honoraria from Abbott Diagnostics, Siemens Healthineers, Roche Diagnostics, and LumiraDx. These companies had no involvement in this article. The remaining authors have no disclosures to report.

## Supporting information

Data S1
Tables S1–S4
References 13, 14, 15, 16, 17, 18
