# Correction to: Infective Endocarditis Hospitalizations and Outcomes in Patients With End‐Stage Kidney Disease: A Nationwide Data‐Linkage Study

**DOI:** 10.1161/JAHA.120.020806

**Published:** 2021-11-24

In the article by Gallacher et al, “Infective Endocarditis Hospitalizations and Outcomes in Patients With End‐Stage Kidney Disease: A Nationwide Data‐Linkage Study”, which published online September 28, 2021 and was included in the October 5, 2021 issue of the Journal (*J Am Heart Assoc*. 2021;10:e022002. DOI: 10.1161/JAHA.121.022002), a correction was needed.

The incorrect publication agreement was selected prior to publication. The article was published under a Creative Commons Attribution‐NonCommercial License, but to comply with a funding mandate, should have been published under the terms of the Creative Commons Attribution License. The license has now been corrected and the statement now reads: “© 2020 The Authors. Published on behalf of the American Heart Association, Inc., by Wiley. This is an open access article under the terms of the Creative Commons Attribution License, which permits use, distribution and reproduction in any medium, provided the original work is properly cited.”

The authors regret the error.

The online version of the article has been updated and is available here: https://www.ahajournals.org/doi/10.1161/JAHA.121.022002.

