# Supplementary material for: Infective Endocarditis Hospitalizations and Outcomes in Patients With End‐Stage Kidney Disease: A Nationwide Data‐Linkage Study
Source: J Am Heart Assoc. 2021 Sep 28;10(19):e022002. doi: 10.1161/JAHA.121.022002 (PMC8649148; doi:10.1161/JAHA.121.022002)
Supplement: Supplementary file 1 — Data S1 Tables S1–S4 References 13, 14, 15, 16, 17, 18 [file JAH3-10-e022002-s001.pdf]

SUPPLEMENTAL MATERIAL

Data S1.

Supplemental Methods

Data sources

Scottish Morbidity Record 01 (SMR01)

The Scottish Morbidity Record 01 (SMR01) is an episode-based hospitalization record, indexed by community health index (CHI) number, relating to all inpatient and day case hospitalizations from non-obstetric and non-psychiatric specialties.[13] A record is generated when a patient completes an episode of inpatient or day case care. Up to six diagnoses are recorded using the International Classification of Diseases (ICD) classification, whilst interventions or procedures are recorded using the OPCS-4 classification. SMR01 data are considered amongst the best routinely-collected healthcare data worldwide in terms of granularity, population coverage and linkage capabilities. Across all SMR01 records, estimated completion and accuracy rates are 99% and 89%, respectively.[14] For SMR01 records relating to cardiovascular diagnoses, the accuracy rate is 94.2%.[9]

National Records of Scotland (NRS) Death Records

The NRS register is indexed by CHI number and records all deaths in Scotland, with ~55,000 deaths registered annually. Available data from individual patients include demographics, date of death, and primary and secondary causes of death.[15] These data were linked to patients identified as having a record of an incident IE hospitalization during the study period. Of note, it is a statutory requirement that any death occurring in Scotland, or out-with Scotland but within the United Kingdom, is registered on the NRS death register within 8 days of death. Although patients who emigrate to other countries will be lost to follow-up, the Scottish population is historically very stable, with a low rate (<0.5%) of overseas emigration each year.[16]

Electronic Communication of Surveillance in Scotland (ECOSS)

The Scottish microbiology surveillance registry or 'Electronic Communication of Surveillance in Scotland' (ECOSS), as it is termed by NHS National Services Scotland, was used to identify positive blood culture results from microbiology laboratories within NHS Scotland health boards pertaining to incident IE hospitalizations in SMR01 between 01/01/2008 and 12/31/2014. Causative organisms were defined as those identified ≤90 days on either side of the incident IE hospitalization date. Polymicrobial status was defined when >1 causative organism was identified on the same culture date. If >1 causative organism was identified on different dates ≤90 days on either side of the incident IE hospitalization date, then the organism identified closest to the incident IE hospitalization date was assigned as the causative organism.

Although data were first recorded in ECOSS from 2007, near-complete data are available from 2008 and so ECOSS records were linked to SMR01 in the present study from this year onwards.[17,18] ECOSS is maintained by NHS National Services Scotland on behalf of Health Protection Scotland. NHS National Services Scotland monitors the completeness and accuracy of ECOSS data through its 'Data Monitoring and Support Service'.[17] Further, NHS National Services Scotland routinely informs data users of any problems affecting the accuracy of these data. More information on ECOSS is available from https://www.hps.scot.nhs.uk/data/.

Identification of study participants

Incident hospitalizations with infective endocarditis (IE), end-stage kidney disease (ESKD) status and comorbidities were defined from SMR01 in patients aged ≥20 years admitted to any Scottish hospital between 01/01/1990 and 12/31/2014 using International Classification of Diseases (ICD) codes (**Table S1**). To optimize specificity and sensitivity, we included only hospitalizations with a diagnostic code for IE appearing in the first 2 (of 6) positions of the SMR01 record. We extracted demographic data (age, sex and deprivation status [see next section]) and selected comorbidities (history of stroke, heart failure, myocardial

infarction, cardiac device and previous cardiac valvular surgery) from SMR01 using records of hospitalizations and procedures during the 5 years preceding hospital admission (a 5-year 'look-back' period). Patients with ESKD were identified by searching linked inpatient records prior to hospitalization with IE for relevant ICD codes (**Table S1**) appearing in any of the 6 available diagnostic positions.

Definition of deprivation status: the Scottish Index of Multiple Deprivation (SIMD)

The Scottish Index of Multiple Deprivation (SIMD) is a geographical-based measure of deprivation. SIMD identifies small geographical regions (where each region is determined by zip code and corresponds to ~750 residents) of material deprivation based on information derived from seven domains (income; employment; health; education, skills and training; geographic access to services; crime; and housing).[6] Each domain is weighted according to its relative importance and provides a score which is then summed with the other domains. The total score for each geographical region enables the areas to be ranked. SIMD scores and ranks (quintiles/deciles) have been used extensively in published epidemiological research from Scotland.[6] In this study, all patients were assigned a SIMD quintile based on their individual SIMD rank at the time of incident IE hospitalization.

Description of look-back period

Table S2 illustrates the look-back period for the years 2000-2010 in three exemplar patients (patients A, B and C). The total incident count for each year is shown in the final column. Where a patient is hospitalized with an episode of IE, a '1' appears in the 'Admission' column. If no IE event has occurred in the 5-years prior (light grey shading), then the event is considered an incident event and a '1' will also appear in the 'Incident' column (dark grey shading).

Table S1. List of International Classification of Diseases codes employed in study to identify incident cases of infective endocarditis, patients with end-stage kidney disease and their comorbidities. Abbreviations: ICD – International Classification of Diseases.

	**Relevant ICD codes**
**Infective endocarditis**	
ICD-9	421.1, 424.91, 424.90, 424.99
ICD-10	I33, I38, I39
**End-stage kidney disease**	
ICD-9	V45.1, V45.11, V56.0 - V56.2, V56.8
ICD-10	Z49.0 - Z49.2, Z94.0, Z99.2
**Myocardial infarction**	
ICD-9	413
ICD-10	I21, I22
**Stroke**	
ICD-9	430 - 438
ICD-10	I60-I69
**Heart failure**	
ICD-9	428
ICD-10	I50
**Cardiac valvular surgery**	
OPCS-4	K04 - K12, K14, K17 - K34
**Cardiac devices**	
OPCS-4	K59 - K61

Table S2. Schematic of the five-year look-back period employed in this study in 3 fictional patients (A, B and C).

Year	Patient A		Patient B		Patient C		Total incident events
	Admission	Incident	Admission	Incident	Admission	Incident	
**2000**	0	0	0	0	0	0	**0**
**2001**	0	0	1	1	0	0	**1**
**2002**	0	0	0	0	0	0	**0**
**2003**	0	0	0	0	0	0	**0**
**2004**	0	0	0	0	0	0	**0**
**2005**	1	1	0	0	1	1	**2**
**2006**	1	0	0	0	0	0	**0**
**2007**	0	0	0	0	1	0	**0**
**2008**	0	0	1	1	0	0	**1**
**2009**	1	0	0	0	0	0	**0**
**2010**	0	0	0	0	0	0	**0**

Table S3. Clinical characteristics of patients with and without end-stage kidney disease hospitalized with infective endocarditis in Scotland between 2008 and 2014, and for whom microbiology data were available. *counts of ≤5 are redacted in line with regulatory approvals to protect patient confidentiality. Abbreviations: ESKD – end-stage kidney disease; SIMD – Scottish Index of Multiple Deprivation.

	**ESKD**	**No ESKD**
**Number of hospitalizations, n (%)**	**81 (3.6)**	**2,186 (96.4)**
**Age, years (SD)**	59.7 (13.1)	66.8 (17.8)
**Sex, n (%)**		
Men	52 (64.2)	1,138 (52.1)
Women	29 (35.8)	1,048 (47.9)
**SIMD quintile, n (%)**		
1 (most deprived)	20 (24.7)	507 (23.2)
2	23 (28.4)	497 (22.7)
3	22 (27.2)	435 (19.9)
4	7 (8.6)	379 (17.3)
5 (least deprived)	8 (9.9)	357 (16.3)
**Previous medical conditions, n (%)**		
Myocardial infarction	6 (7.4)	107 (4.9)
Stroke	7 (8.6)	105 (4.8)
Heart failure hospitalization	NA*	254 (11.6)
Cardiac device	NA*	53 (2.4)
Prior cardiac valve surgery	NA*	167 (7.6)
**Microbiology, n (%)**		
Staphylococcus aureus	21 (25.9)	280 (12.8)
Coagulase-negative *Staphylococcus* spp.	8 (9.9)	94 (4.3)
Streptococcus spp.	8 (9.9)	329 (15.1)
Other (polymicrobial/*Enterococcus* spp.)	10 (12.3)	200 (9.1)
Negative blood cultures/blood cultures not taken	34 (42.0)	1,283 (58.7)

Table S4. Odds ratios and 95% confidence intervals from logistic regression model for outcome of death at 1 and 3 years in patients with end-stage kidney disease (ESKD) only. The model adjusted for age, sex, deprivation status and history of stroke, heart failure hospitalization and myocardial infarction. Abbreviations: CI – confidence interval; SIMD – Scottish Index of Multiple Deprivation.

	**Death at 1 year**				**Death at 3 years**			
	**Odds ratio**	**Lower 95% CI**	**Upper 95% CI**	**p-value**	**Odds ratio**	**Lower 95% CI**	**Upper 95% CI**	**p-value**
**Patient demographics**								
Age, per 10 year increase	1.34	1.09	1.67	0.006	1.44	1.16	1.81	0.001
Female sex	0.77	0.43	1.38	0.379	1.03	0.56	1.90	0.928
**SIMD quintile** **(*1 = most deprived; 5 = least deprived*)**								
2 (*vs*. quintile 1)	0.79	0.35	1.76	0.565	1.07	0.45	2.55	0.880
3 (*vs*. quintile 1)	0.65	0.28	1.48	0.308	0.51	0.21	1.19	0.124
4 (*vs*. quintile 1)	1.51	0.56	4.18	0.418	1.52	0.50	5.01	0.470
5 (*vs*. quintile 1)	0.38	0.13	1.01	0.059	0.34	0.12	0.92	0.037
**Comorbidities**								
Stroke	0.71	0.21	2.13	0.552	0.75	0.24	2.38	0.612
Heart failure hospitalisation	0.77	0.29	1.96	0.593	2.01	0.71	6.68	0.213
Myocardial infarction	1.06	0.31	3.41	0.920	1.46	0.42	5.90	0.563
